# Australian general practice registrars’ billing patterns: a cross-sectional analysis from the Registrars Clinical Encounters in Training (ReCEnT) study

**DOI:** 10.1186/s12913-024-11834-y

**Published:** 2024-11-27

**Authors:** Katie Fisher, Amanda Tapley, Anna Ralston, Andrew Davey, Elizabeth Holliday, Jason Dizon, Susan Wearne, Alison Fielding, Mieke van Driel, Neil Spike, Lisa Clarke, Parker Magin

**Affiliations:** 1 University of Newcastle, School of Medicine and Public Health, University Drive, University Drive, Callaghan, NSW 2308 Australia; 2 GP Training, Training Research Department, Royal Australian College of General Practitioners (RACGP), Level 1, 20 McIntosh Drive, Mayfield West, NSW 2304 Australia; 3 Hunter Medical Research Institute, Clinical Research Design, IT and Statistical Support Unit (CReDITSS), New Lambton Heights, 2305 Australia; 4 Department of Health, Canberra, ACT 2601 Australia; 5 School of Medicine and Psychology, Australian National University, Academic Unit of General Practice, Canberra, ACT 2601 Australia; 6 Faculty of Medicine, General Practice Clinical Unit, University of Queensland, Royal Brisbane & Women’s Hospital, Level 8 Health Sciences Building, Brisbane, QLD 4029 Australia; 7 Department of General Practice and Primary Health Care, University of Melbourne, Berkeley Street, Carlton, VIC 3053 Australia; 8 School of Rural Health, Faculty of Medicine, Nursing and Health Sciences, Monash University, Clayton, VIC 3168 Australia; 9 Medical Education Department, Royal Australian College of General Practitioners (RACGP), GP Training, 62 Patrick Street, Hobart, TAS 7000 Australia

**Keywords:** General practice, Education, Medical, Graduate, Fees, Medical

## Abstract

**Background:**

In Australia, a government insurance scheme (Medicare) pays set rebates for a range of distinct general practitioner (GP) services. GPs may ‘bulk-bill’ and accept the Medicare rebate fee directly, or ‘privately-bill’ by charging the patient a higher fee that is partially reimbursed by Medicare. The billing behaviour of Australian GP registrars (trainees) and their decision to bulk- or privately-bill patients is an evidence gap. This study aimed to establish the prevalence and associations of registrars’ bulk-billing versus private-billing.

**Methods:**

A cross-sectional analysis of data from the ReCEnT study, 2010–2021. The primary analysis used univariable and multivariable logistic regression, with the outcome factor being whether a consultation was bulk-billed versus privately-billed. The primary analysis excluded practices that universally bulk-bill or universally privately-bill all patients. A secondary analysis included all practices regardless of billing policy to provide an overall perspective of billing across the breadth of GP vocational training.

**Results:**

For the primary analysis, 3,086 GP registrars recorded details of 316,141 consultations. Bulk-billing accounted for 61.8%, [95% CI:61.6%, 62.0%] of consultations. Significant positive associations of bulk-billing included: younger and older patient age (compared to patients aged 15–34 years, aOR 5.45; CI: [5.06, 5.87] for patients aged 0–14 years, aOR 2.36; 95% CI: [2.24, 2.49] for patients aged 65–74 years, and aOR 4.48; CI: [4.13, 4.85] for 75 years-and-older). Significant negative associations of bulk-billing included patients new to the practice (aOR 0.39; CI: [0.37, 0.41]) and patients new to the registrar (aOR 0.56; CI: [0.55, 0.58]), compared to existing patients of the registrar and practice; and practices with lesser socio-economic disadvantage (aOR 0.91; CI: [0.89, 0.93] per decile decrease in socioeconomic disadvantage). Bulk-billed consultations were positively associated with arranging patient follow-up (with the registrar aOR 1.06; CI: [1.03, 1.09]; or with another GP in the practice aOR 1.40; CI: [1.33, 1.46]).

**Conclusions:**

Registrar billing decisions may, in part, reflect government bulk-billing incentives but our findings suggest other factors may contribute, including the provision of affordable care recognising patient need (children and elderly, and those living in areas of greater socioeconomic disadvantage) and continuity of care. Further research is needed to better understand how, and why, registrars make billing decisions.

**Supplementary Information:**

The online version contains supplementary material available at 10.1186/s12913-024-11834-y.

## Background

General practice (family medicine) is the cornerstone of primary healthcare systems, contributing to improved quality of care, lowering all-cause mortality, and lowering total healthcare system costs [1]. To achieve these outcomes, general practice requires appropriate funding. The increasing prevalence of chronic health conditions is resulting in longer and more complex consultations [2] and general practitioners report that the cost of care is increasing [3]. 

In Australia, general practice is funded by a mixed model. This is primarily comprised of the Medicare Benefits Schedule (MBS), which is Australia’s public health insurance scheme, and operates on a fee-for-service model, with set rebates for a wide range of individual clinical services [4]. General practitioners (GPs) may ‘bulk-bill’ and accept the applicable Medicare rebate fee directly, or ‘privately-bill’ by charging the patient a higher fee that is partially reimbursed by Medicare [4]. While the rebate is ‘set’, there are modest incentives for bulk-billing patients from specific demographics (children under 16 years old, pensioners, and low-income concession-card holders) [5]. However, it should be noted that these incentive payments are well below the average out-of-pocket fee for a mixed-billing practice (average out-of-pocket being A$40.55 in 2022) [6]. 

GP registrars (vocational specialist trainees in general practice) make up 13% of the Australian GP workforce by headcount [7]. While GPs may find the MBS complex, it can be particularly challenging for registrars, most of whom have no prior experience in billing to Medicare. In addition, though having the same billing rights as established GPs, GP registrars may be hesitant to privately-bill patients due to their ‘in-training’ status. The GP Supervisors Australia website notes: “Registrars may ‘undersell’ themselves and fail to bill appropriately – it is important to value the service provided despite relative inexperience” [8]. Wearne et al. also describe Medicare billing as a “craft” of general practice that must be discussed between registrars and supervisors [9]. 

There is limited literature available on established GPs’ billing practices in Australia. According to Medicare statistics, from October-December 2022, 80.5% of general practice services were bulk-billed [10]. However, this figure is misleading as it refers to the number of ‘services’, not consultations, that were bulk-billed; it is possible to co-bill several ‘services’ (or item numbers) in one consultation. By contrast, the 2022 Royal Australian College of General Practitioners’ (RACGP) Health of the Nation survey found that only 24% of GPs bulk-bill all of their patients [6]. This is in the context of a long-term decline in the financial viability of Australian general practice, exacerbated by frozen indexation for Medicare rebates for GP services during financial years 2013-14, and 2015-16 to 2017-18 inclusive [11]. 

The billing patterns of GP registrars are of particular importance to Australian GP vocational training, as remuneration via fee-for-service largely determines both registrars’ and teaching practices’ incomes. In summary, most Australian GP registrars are employed under a contract, where they are paid a base rate of pay, with a ‘top-up’ payment, being the difference between their base rate and a percentage of their billings (with the practice receiving the remaining percentage). This has implications for recruitment of junior doctors to GP vocational training [12, 13] and also affects the financial viability for practices of taking on registrar training [14, 15]. The effect on attractiveness to junior doctors of training in general practice is particularly relevant when considering the projected workforce shortfall of 11,392 full-time GPs in Australia by 2032 [16]. 

The aims of this study were to establish the prevalence and (registrar, patient, practice, and consultation) associations of Australian GP registrars’ bulk-billing versus private billing. And, thus, to inform policy of relevant stakeholders, including teaching practices and training providers.

## Methods

This was a cross-sectional analysis of data from the Registrar Clinical Encounters in Training (ReCEnT) project [17]. ReCEnT is an ongoing multi-site inception cohort study of GP registrars’ in-consultation clinical and educational experience and behaviours [18, 19]. The detailed study methodology has been published, but briefly: Australian GP registrars undergo at least three 6-month, full-time-equivalent, terms in community-based general practice. In ReCEnT, registrars record data from 60 consecutive office-based consultations in each of three 6-monthly terms, documenting the clinical and educational details of the consultation. Nursing home consultations, home visits, consultations in Aboriginal Medical Services, and consultations in ‘single-purpose clinics’ (e.g., immunisation clinics) are not included. From 2010–2015, ReCEnT was conducted in five of 17 Regional Training Providers (RTPs) across five states (New South Wales, Victoria, Queensland, South Australia, and Tasmania), and from 2016–2022, in three of nine Regional Training Organisations across three states (New South Wales, Victoria, Tasmania) and the Australian Capital Territory.

### Outcome factor

The outcome factor was consultations bulk-billed versus privately-billed. GP registrars are asked to record the billing status of the consultation and can select one or more of the following options: “private”, “bulk-bill”, “no charge”, “workers compensation”, and “other”.

### Inclusion criteria

*Primary analysis*: mixed billing practices (mix of bulk-billing and private-billing).

*Secondary analysis*: all practices (those that universally bulk-bill, those that universally private bill, and mixed billing practices).

Consultations with multiple billing types selected by the registrar for individual items (multiple items can be individually billed within the one consultation) were coded as follows:

- Consultation coded as privately-billed if the registrar selected ‘private’ along with any of the following billing types: ‘bulk-bill’, ‘no charge’ and/or ‘other’.
- Consultation coded as bulk-billed if the registrar selected ‘bulk-bill’ along with any of the following billing types: ‘no charge’ and/or ‘other’.

### Exclusion criteria – both analyses

- Consultations billed as only “workers compensation”, “no charge”, or “other”.

### Exclusion criteria – primary analysis

- Practices with missing billing information.
- Practices that universally bulk-bill all patients or universally privately-bill all patients.
- All telehealth consultations, given compulsory bulk-billing for these item numbers between 13/03/2020 and 06/04/2020, and further restrictions on private billing from 06/04/2020 to 01/10/2020 for concession card holders, children under 16 and patients more vulnerable to COVID-19.
- Consultations including chronic disease item numbers (GP Management Plans, Team Care Arrangements, and Health Assessments), as these are typically bulk-billed (excluded item numbers 721, 723, 729, 731, 732, 701, 703, 705, and 707).

### Independent variables

Independent variables included in the analyses are shown in Table 1. Independent variables were selected by GP researchers (KF, PM) based on their potential to influence billing decisions.

**Table 1:** Independent variables included in analyses

**Time variable**	• Year
**Registrar variables**	• Age • Gender • Training term • Place of medical qualification (Australia/international) • Full-time/part-time status • Whether the registrar had previously worked at the practice • Whether the registrar had prior health qualifications or post-graduate qualifications • Whether the registrar does work outside of GP training
**Patient variables**	• Age • Gender • Aboriginal or Torres Strait Islander status • Non-English-speaking background • Patient practice status (whether the patient is new to the registrar, whether the patient is new to the practice, or whether the patient is known to both the registrar and practice)
**Practice variables**	• Rurality/urbanicity, as determined by the Australian Statistical Geography Standard Remoteness Area (ASGS-RA) • Practice size (full-time equivalent GPs) • Practice location’s Socio-Economic Indexes for Areas– Index of Relative Socioeconomic Disadvantage (SEIFA-IRSD) • Regional training provider (RTP)
**Consultation variables**	• Number of problems addressed in the consultation • Consultation duration • Problem status of consultation (only existing problems, only new problems, or both existing and new problems) • Whether assistance or advice was sought • Medications prescribed • Imaging ordered • Pathology ordered • Follow-up organised • Referrals made • Learning goals generated

### Statistical analysis

We conducted cross-sectional analysis using data from 24 rounds of 6-monthly collected data from 2010 to 2021.

Associations between independent variables and the outcome of a consultation being bulk-billed were estimated using univariable and multivariable logistic regression. Logistic regression was used within the generalised estimating equations framework to adjust for repeated measures within registrars. An exchangeable working correlation structure was assumed. Descriptive statistics included frequencies for categorical variables and mean with standard deviation for continuous variables. Missing data was managed using a complete case analysis approach.

As there have been considerable shifts in financial stresses upon GPs and subsequent attitudes to bulk/private billing during data collection (2010–2021), the variable ‘year’ was entered as an independent variable in our analyses. This variable was included in continuous form, assuming a linear relationship with the log-odds of bulk-billing (as indicated by the data). In our secondary analysis (see below) this also allowed us to evaluate temporal trends in overall use of bulk-billing by registrars.

Multivariable analyses were conducted including all explanatory variables of interest. Variables in the model with *p* values > 0.2 were tested for removal. A variable was removed if the resulting model did not have substantively different effect sizes than the previous model. A substantive change in effect size (beta coefficient) was defined as greater than 10%.

A secondary analysis was performed including all practices and all item numbers, using the same statistical methods. The rationale for the primary and secondary analyses was that the primary analysis explored associations of bulk-bill volitional decision-making by the registrar regarding billing (that is, when this decision-making was not dictated by a blanket policy at the practice level). The secondary analysis explored associations of bulk-billing during the totality of registrar practice – which provides an overall perspective of bulk-billing and non-bulk-billing across the breadth of GP vocational training. This has implications for registrar remuneration (and, to some extent, relative attractiveness of GP vocational training) and for the financial viability of registrar training for teaching practices.

The regressions modelled the log-odds that the consultation was bulk-billed rather than privately-billed. Effects were expressed as adjusted odds ratios (aORs) with 95% confidence intervals (CI). Significance was declared at the conventional 0.05 level, with the magnitude and precision of effect estimates also used to interpret results.

Statistical analyses were programmed using STATA 16.0 (StataCorp, College Station, TX, USA) and SAS V9.4 (SAS Institute Inc., Cary, NC, USA).

### Ethics approval

This study has ethics approval from the University of Newcastle Human Research Ethics Committee Reference H-2009-0323.

## Results

Between 2010 and 2021, 3,850 GP registrars recorded details of 548,006 consultations (response rate 92.2%). Of these, 30.4% were from practices that universally bulk-bill patients, 2.7% were from practices that universally privately-bill patients, 62.2% were from mixed-billing practices, and 4.7% had missing billing information. Table 2 presents the demographic characteristics of the participating GP registrars.

**Table 2:** Demographics of participating GP registrars (*n* = 3,850 registrars, 2010–2021)

	Variables	Class	Total *n* (%)
**Registrar characteristics (*n*** **= 3850)**	Registrar gender	Male	1497 (38.88)
	Has Australian medical degree	Yes	3057 (80.70)
	Year of graduation	Mean (SD^a^)	2010.76 (5.74)
	Pathway registrar enrolled in	General	2593 (68.25)
	Has post-graduate qualifications	Yes	1203 (31.74)
	College seeking fellowship	RACGP^b^	3590 (96.45)
		ACCRM^c^	98 (2.63)
		Both	34 (0.91)
**Registrar-round/practice characteristics (*n*** **= 9755)**	Registrar age (years)	Mean (SD)	32.72 (6.23)
	Registrar works part-time	Yes	2139 (23.08)
	Registrar training term	Term 1	3671 (37.63)
		Term 2	3429 (35.15)
		Term 3	2655 (27.22)
	Registrar does other medical work	Yes	1603 (17.56)
	Registrar worked at practice previously	No	7097 (75.71)
	Size of practice	Small (≤ 5 FTE GPs)	3651 (39.44)
	Rurality of practice	Major city	5870 (60.78)
		Inner regional	2790 (28.89)
		Outer regional	920 (9.52)
		Remote	62 (0.64)
		Very remote	16 (0.17)
	SEIFA-IRSD^d^ decile of practice	Mean (SD)	5.37 (2.80)

^a^ Standard deviation

^b^ Australian College of Rural and Remote Medicine

^c^ Royal Australian College of General Practitioners

^d^ Socio-Economic Indexes for Areas for Disadvantage – Index of Relative Socioeconomic Disadvantage (a low decile indicates relatively greater disadvantage and a high decile indicates a relative lack of disadvantage)

### Primary analysis: mixed-billing practices

For the primary analysis, including only mixed-billing practices, 3,086 GP registrars recorded 316,141 consultations. Bulk-billing accounted for 61.8% of consultations, CI: [61.6%, 62.0%]. Table S1 (Supplementary) presents the characteristics associated with bulk-billing, Table S2 presents data missingness, and Table 3 presents the univariable and multivariable (adjusted) associations.

**Table 3:** Associations with bulk-billing versus private-billing (primary analysis; *n* = 3,086 GP registrars, *n* = 316,141 consultations, 2010–2021)

			Univariable		Adjusted	
Variable group	Variable	Class	OR [95% CI]	*P* value	OR [95% CI]	*P* value
Time variable	Year		1.05 (1.03, 1.07)	< 0.001	1.00 (0.98, 1.02)	0.76
Patient variables	Patient age group Referent: 15–34 years	0–14 years	4.35 (4.11, 4.61)	< 0.001	5.45 (5.06, 5.87)	< 0.001
		35–64 years	0.86 (0.84, 0.87)	< 0.001	0.78 (0.76, 0.80)	< 0.001
		65–74 years	2.35 (2.25, 2.44)	< 0.001	2.36 (2.24, 2.49)	< 0.001
		75 + years	4.41 (4.15, 4.68)	< 0.001	4.48 (4.13, 4.85)	< 0.001
	Patient gender Referent: Male	Female	0.93 (0.91, 0.95)	< 0.001	1.07 (1.05, 1.10)	< 0.001
	Patient/practice status Referent: Known to the registrar and the practice	New to registrar	0.59 (0.58, 0.61)	< 0.001	0.56 (0.55, 0.58)	< 0.001
		New to practice	0.41 (0.39, 0.42)	< 0.001	0.39 (0.37, 0.41)	< 0.001
	NESB^a^	Yes	1.15 (1.08, 1.21)	< 0.001	1.17 (1.08, 1.23)	< 0.001
	Aboriginal or Torres Strait Islander status	Yes	2.69 (2.49, 2.92)	< 0.001	3.17 (2.83, 3.55)	< 0.001
Registrar variables	Registrar gender Referent: Male	Female	0.85 (0.79, 0.91)	< 0.001	0.85 (0.77, 0.94)	0.002
	Registrar FTE^b^ Referent: Full-time	Part-time	0.88 (0.81, 0.96)	0.005	0.94 (0.84, 1.04)	0.24
	Training term/post Referent: Term 1	Term 2	1.05 (1.00, 1.10)	0.059	1.04 (0.95, 1.14)	0.43
		Term 3	1.02 (0.96, 1.09)	0.50	1.04 (0.94, 1.15)	0.45
	Qualified as doctor in Australia	Yes	0.91 (0.83, 1.00)	0.052	0.84 (0.71, 0.99)	0.037
	Worked at practice previously	Yes	1.10 (1.06, 1.15)	< 0.001	0.99 (0.91, 1.08)	0.80
	Previous qualifications	Health	1.06 (0.95, 1.18)	0.28	1.01 (0.86, 1.18)	0.90
		Non-health	0.98 (0.91, 1.07)	0.71	1.00 (0.89, 1.12)	0.99
	Post-graduate qualifications	Yes	0.97 (0.90, 1.05)	0.43	0.96 (0.86, 1.06)	0.40
	Has other regular medical work	Yes	1.03 (0.94, 1.12)	0.55	1.02 (0.91, 1.13)	0.74
	Registrar age		1.00 (0.99, 1.01)	0.60	0.99 (0.98, 1.00)	0.30
Practice variables	Practice size Referent: Large (> 5 GPs)	Small (1–5 GPs)	1.13 (1.05, 1.22)	0.002	1.18 (1.07, 1.29)	< 0.001
	Rurality Referent: Urban	Inner regional	1.11 (0.97, 1.27)	0.14	1.10 (0.91, 1.33)	0.33
		Outer regional remote	1.42 (1.18, 1.70)	< 0.001	1.14 (0.89, 1.47)	0.31
	Region Referent: Region 1	Region 2	0.84 (0.71, 0.99)	0.043	0.57 (0.44, 0.72)	< 0.001
		Region 3	0.93 (0.80, 1.08)	0.33	0.52 (0.42, 0.64)	< 0.001
		Region 4	0.83 (0.73, 0.94)	0.003	0.73 (0.63, 0.84)	< 0.001
		Region 5	0.89 (0.70, 1.13)	0.34	0.47 (0.33, 0.67)	< 0.001
		Region 6	1.76 (1.34, 2.31)	< 0.001	2.52 (1.96, 3.24)	< 0.001
		Region 7	0.93 (0.78, 1.11)	0.43	0.71 (0.60, 0.85)	< 0.001
	SEIFA-IRSD^c^		0.92 (0.90, 0.94)	< 0.001	0.91 (0.89, 0.93)	< 0.001
Consultation variables	Problem status Referent: Only existing problems	Both existing and new problems	0.68 (0.66, 0.69)	< 0.001	0.77 (0.75, 0.80)	< 0.001
		Only new problem	0.66 (0.64, 0.67)	< 0.001	0.67 (0.66, 0.69)	< 0.001
	Sought assistance Referent: None	Other sources	0.93 (0.91, 0.96)	< 0.001	0.93 (0.91, 0.96)	< 0.001
		Supervisor	1.02 (0.99, 1.05)	0.14	1.03 (0.99, 1.07)	0.12
	Consultation duration		0.99 (0.99, 0.99)	< 0.001	1.00 (1.00, 1.00)	0.001
	Number of problems		0.92 (0.91, 0.93)	< 0.001	0.98 (0.96, 1.00)	0.019
	Medication prescribed	Yes	0.92 (0.90, 0.93)	< 0.001	0.93 (0.91, 0.95)	< 0.001
	Imaging ordered	Yes	0.71 (0.70, 0.73)	< 0.001	0.83 (0.81, 0.85)	< 0.001
	Pathology ordered	Yes	0.63 (0.62, 0.64)	< 0.001	0.77 (0.75, 0.79)	< 0.001
	Referral ordered	Yes	1.03 (1.01, 1.05)	0.003	1.01 (0.99, 1.04)	0.37
	Follow-up ordered Referent: None	Other GP in the practice	1.31 (1.26, 1.35)	< 0.001	1.40 (1.33, 1.46)	< 0.001
		With themselves	0.95 (0.93, 0.97)	< 0.001	1.06 (1.03, 1.09)	< 0.001
	Learning goals generated	Yes	0.90 (0.88, 0.92)	< 0.001	0.94 (0.91, 0.97)	< 0.001

^a^ Non-English speaking background

^b^ Full-time equivalent

^c^ Socio-Economic Indexes for Areas – Index of Relative Socio-Economic Disadvantage

Notable associations of independent variables included:

For patient variables, bulk-billing was significantly associated with extremes of age. Children aged 0–14 years had five times the odds (aOR 5.45; CI: [5.06, 5.87]; *p* < 0.001), patients aged 65–74 years had two times the odds (aOR 2.36; CI: [2.24, 2.49]; *p* < 0.001); and patients aged 75 years and older had four times the odds (aOR 4.48; CI: [4.13, 4.85]; *p* < 0.001) of being bulk-billed compared to those aged 15–34 years. Female patients (aOR 1.07; CI: [1.05, 1.10]; *p* < 0.001) and non-English speaking background (NESB) patients (aOR 1.15; CI: [1.08, 1.23]; *p* < 0.001) had somewhat higher odds of being bulk-billed compared to males or English-speaking background patients, respectively. However, Aboriginal and/or Torres Strait Islander patients had three times the odds of being bulk-billed (aOR 3.17; CI: [2.83, 3.55]; *p* < 0.001) compared to non-Aboriginal and/or Torres Strait Islander patients.

Compared to existing patients (known to the practice and the registrar), there were significantly lower odds of bulk-billing for patients new to the practice and registrar, (aOR 0.39; CI: [0.37, 0.41]; *p* < 0.001); and for patients new to the registrar but known to the practice (aOR 0.56; CI: [0.55, 0.58]; *p* < 0.001).

For practice variables, bulk-billing was associated with smaller practice size (1–5 GPs, aOR 1.18; CI: [1.07, 1.29]; *p* < 0.001) and areas of greater socio-economic disadvantage (OR 0.91; CI: [0.89, 0.93] per decile; *p* < 0.001). There was considerable variation in bulk-billing by training region (aORs of 0.47 to 2.52 compared to the referent region). There was no statistically significant association between billing and rurality.

For consultation variables, bulk-billing was associated with addressing only existing problems (compared to only new problems aOR 0.67; CI: [0.66, 0.69]; *p* < 0.001; and both new and existing problems aOR 0.77; CI: [0.75, 0.80]; *p* < 0.001); and higher rates of follow-up (with themselves aOR 1.06; CI: [1.03, 1.09]; *p* < 0.001; with another GP in the practice aOR 1.40; CI: [1.33, 1.46]; *p* < 0.001).

For bulk-billed consultations, there was a negative association with seeking assistance from other sources, including electronic resources, books, and discussions with other healthcare providers (aOR 0.93; CI: [0.91, 0.96]; p = < 0.001); generating learning goals from the consultation (aOR 0.94; CI: [0.91, 0.97]; *p* < 0.001); medicines being prescribed (aOR 0.93; CI: [0.91, 0.95]; *p* < 0.001); and imaging (aOR 0.83; CI: [0.81, 0.85]; *p* < 0.001) or pathology being ordered (aOR 0.77; CI: [0.75, 0.79]; *p* < 0.001).

### Secondary analysis – all practices

For the secondary analysis, which included all practices (regardless of billing status) and all item numbers, 74.0% of consultations were bulk-billed, CI: [73.9%, 74.1%]. Overall, the results were similar between the two analyses, with a few notable differences. Table S3 shows the characteristics associated with bulk-billing, Table S4 presents missingness and Table 4 shows the univariable and multivariable (adjusted) associations.

**Table 4:** Associations with bulk-billing versus private-billing (secondary analysis, *n* = 3,850 GP registrars, *n* = 548,006 consultations, 2010–2021)

			Univariable		Adjusted	
Variable group	Variable	Class	OR [95% CI]	*P* value	OR [95% CI]	*P* value
Time variable	Year		1.03 (1.01, 1.06)	0.019	1.07 (1.04, 1.10)	< 0.001
Patient variables	Patient age group. Referent: 15–34 years	0–14 years	3.00 (2.86, 3.14)	< 0.001	3.85 (3.61, 4.11)	< 0.001
		35–64 years	0.90 (0.89, 0.92)	< 0.001	0.83 (0.81, 0.85)	< 0.001
		65–74 years	1.93 (1.86, 2.00)	< 0.001	2.00 (1.91, 2.10)	< 0.001
		75 + years	3.39 (3.20, 3.60)	< 0.001	3.55 (3.29, 3.84)	< 0.001
	Patient gender. Referent: Male	Female	0.96 (0.95, 0.97)	< 0.001	1.07 (1.05, 1.09)	< 0.001
	Patient/practice status Referent: known to both the registrar and the practice	New to registrar	0.62 (0.60, 0.63)	< 0.001	0.60 (0.58, 0.61)	< 0.001
		New to practice	0.43 (0.42, 0.45)	< 0.001	0.42 (0.40, 0.43)	< 0.001
	NESB^a^	Yes	1.15 (1.11, 1.20)	< 0.001	1.13 (1.06, 1.19)	< 0.001
	Aboriginal and/or Torres Strait Islander	Yes	2.47 (2.30, 2.66)	< 0.001	2.84 (2.57, 3.13)	< 0.001
Registrar variables	Registrar gender Referent: Male	Female	0.80 (0.74, 0.87)	< 0.001	0.82 (0.72, 0.92)	0.001
	Registrar FTE^b^ Referent: Full-time	Part-time	0.91 (0.83, 0.99)	0.025	0.97 (0.87, 1.09)	0.63
	Training term/post Referent: Term 1	Term 2	1.01 (0.96, 1.06)	0.73	0.95 (0.88, 1.04)	0.29
		Term 3	0.92 (0.87, 0.98)	0.012	0.84 (0.76, 0.93)	0.001
	Qualified as doctor in Australia	Yes	0.92 (0.83, 1.02)	0.10	0.74 (0.61, 0.89)	0.002
	Worked at practice previously	Yes	1.09 (1.05, 1.14)	< 0.001	1.04 (0.96, 1.13)	0.35
	Previous qualifications	Health	1.05 (0.94, 1.17)	0.42	0.91 (0.75, 1.10)	0.33
		Non-health	0.98 (0.90, 1.06)	0.56	0.98 (0.84, 1.13)	0.74
	Post-graduate qualifications	Yes	0.98 (0.91, 1.06)	0.62	1.00 (0.88, 1.14)	0.99
	Has other regular medical work	Yes	0.97 (0.88, 1.07)	0.53	0.97 (0.86, 1.10)	0.66
	Registrar age		0.99 (0.98, 1.00)	0.082	1.00 (0.99, 1.01)	0.84
Practice variables	Practice size Referent: Large (> 5 GPs)	Small (1–5 GPs)	1.31 (1.21, 1.40)	< 0.001	1.32 (1.21, 1.45)	< 0.001
	Rurality Referent: Urban	Inner regional	0.79 (0.66, 0.94)	0.007	0.77 (0.63, 0.95)	0.015
		Outer regional remote	0.94 (0.74, 1.18)	0.58	0.70 (0.53, 0.93)	0.013
	Region Referent: Region 1	Region 2	0.88 (0.70, 1.10)	0.26	0.81 (0.62, 1.06)	0.13
		Region 3	0.80 (0.65, 0.98)	0.030	0.63 (0.50, 0.80)	< 0.001
		Region 4	1.13 (0.94, 1.37)	0.20	1.30 (1.10, 1.53)	0.002
		Region 5	0.88 (0.66, 1.16)	0.36	0.80 (0.53, 1.20)	0.28
		Region 6	4.02 (2.78, 5.82)	< 0.001	6.07 (4.64, 7.94)	< 0.001
		Region 7	1.27 (0.97, 1.65)	0.078	1.08 (0.87, 1.35)	0.49
	SEIFA-IRSD^c^		0.89 (0.87, 0.90)	< 0.001	0.86 (0.84, 0.87)	< 0.001
Consultation variables	Problem status Referent: Only existing problems	Both existing and new problems	0.70 (0.68, 0.71)	< 0.001	0.77 (0.75, 0.80)	< 0.001
		Only new problem	0.66 (0.65, 0.67)	< 0.001	0.69 (0.68, 0.71)	< 0.001
	Sought assistance Referent: None	Other sources	0.93 (0.91, 0.95)	< 0.001	0.94 (0.91, 0.96)	< 0.001
		Supervisor	0.99 (0.96, 1.02)	0.58	0.98 (0.95, 1.02)	0.37
	Consultation duration		0.99 (0.99, 0.99)	< 0.001	1.00 (1.00, 1.00)	0.001
	Medication prescribed	Yes	0.90 (0.89, 0.92)	< 0.001	0.90 (0.88, 0.92)	< 0.001
	Imaging ordered	Yes	0.75 (0.73, 0.76)	< 0.001	0.83 (0.81, 0.86)	< 0.001
	Pathology ordered	Yes	0.68 (0.67, 0.69)	< 0.001	0.78 (0.76, 0.80)	< 0.001
	Referral ordered	Yes	1.04 (1.03, 1.06)	< 0.001	1.02 (0.99, 1.04)	0.21
	Follow-up ordered	Other GP in the practice	1.27 (1.23, 1.31)	< 0.001	1.35 (1.29, 1.41)	< 0.001
	Referent: None	With themselves	0.98 (0.97, 1.00)	0.072	1.10 (1.07, 1.13)	< 0.001
	Learning goals generated	Yes	0.92 (0.90, 0.94)	< 0.001	0.96 (0.93, 0.99)	0.004

^a^ Non-English speaking background

^b^ Full-time equivalent

^c^ Socio-Economic Indexes for Areas – Index of Relative Socio-Economic Disadvantage

Firstly, bulk-billing was significantly associated with training term, with lower odds for bulk-billing for Term 3 GP registrars compared to Term 1 (aOR 0.84; CI: [0.76, 0.93]; *p* = 0.001). And secondly, that bulk-billing was negatively associated with inner regional areas (aOR 0.77; CI: [0.63, 0.95]; *p* = 0.015) and outer regional and remote areas (aOR 0.70; CI: [0.53, 0.93]; *p* = 0.013) when compared to urban areas.

**Fig. 1:**
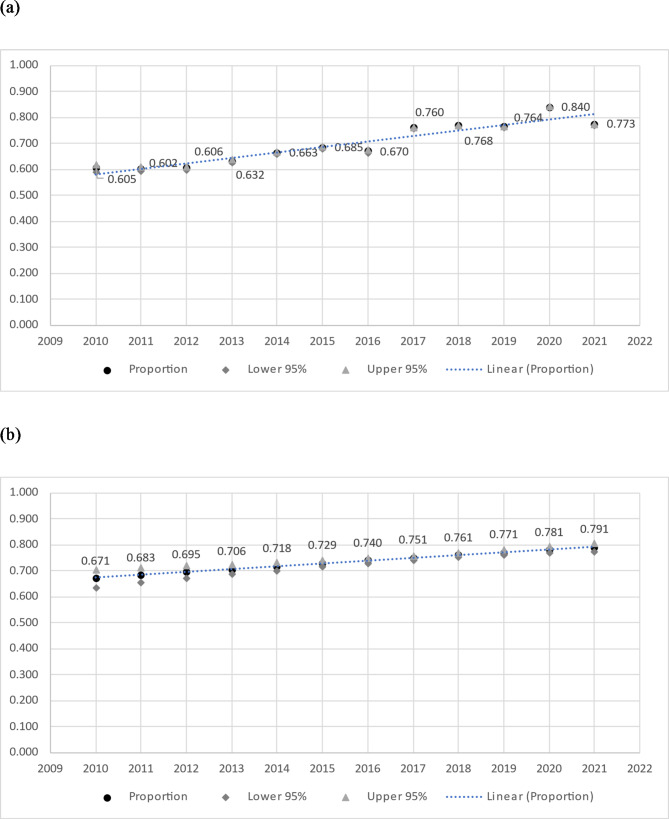
**a** Unadjusted proportion of consultations bulk-billed by year (secondary analysis, *n* = 3,850 GP registrars, *n* = 548,006 consultations, 2010-2021). **b** Adjusted proportion of consultations bulk-billed by year (secondary analysis, *n* = 3,850 GP registrars, *n* = 548,006 consultations, 2010-2021)

There was also an increase in bulk-billing rates during the period 2010–2021, significant both univariably and in the multivariable analysis. See Fig. 1 for graphical presentation of temporal trend in bulk-billing.

## Discussion

### Summary of findings

This exploratory study looked at the associations of GP registrars’ bulk-billing versus private billing for in-practice consultations. Between 2010 and 2021, 61.8% of registrar consultations in mixed-billing practices were bulk-billed.

Of particular interest, bulk-billing was positively associated with the patient being < 15 years old or > 64 years old, female, non-English speaking background, Aboriginal and/or Torres Strait Islander, and with the patient being an existing patient of the practice. Bulk-billed consultations were also positively associated with arranging a follow-up consultation (especially with another doctor) and addressing only existing problems. These findings may be attributable, in part, to government bulk-billing incentives and practice billing policies, however, there do appear to be findings attributable to registrar volition beyond these factors.

### Comparison to existing literature and interpretation of findings

These findings suggest that a patient’s ‘capacity to pay’, and their level of need, influences GP registrars’ billing decisions. Certain populations may have reduced capacity to pay out-of-pocket and/or greater medical need, including those with greater socioeconomic disadvantage [20], older Australians [21], Aboriginal and/or Torres Strait Islanders [22], and patients from NESB [23]. For example, one 2022 study found that 70% of women from non-English speaking backgrounds and 53% of Aboriginal and/or Torres Strait Islander women couldn’t afford to see a health professional when they needed it [23]. Some of these groups are also more likely to be concession- and pension card-holders, which could influence the decision to bulk-bill due to government incentives [5] as well as concerns for the patient’s capacity to pay. It is worth noting that these patient groups may be eligible for chronic disease management (CDM) item numbers, which attract a higher Medicare rebate. While these item numbers were excluded from the primary analysis, a patient’s eligibility for these services may influence billing decisions.

A practice being located in an area of greater socioeconomic disadvantage was strongly associated with bulk-billing. Patients residing in these areas may have reduced capacity to pay out-of-pocket fees [24, 25]. In terms of rurality, our primary analysis showed no significant association between billing and rurality for mixed billing practices. By contrast, our secondary analysis, including practices that universally bulk-bill, showed a significant positive association between urban regions and bulk-billing, compared to inner regional and outer regional/remote locations. This is in keeping with existing literature [24–26]. 

Our findings suggest that continuity of care is associated with-bulk-billing, in that GP registrars had higher odds of bulk-billing a consultation for existing patients known to the practice and if a follow-up consultation was scheduled. Furthermore, GP registrars had higher odds of bulk-billing a consultation addressing only existing problems (which will often involve CDM). Continuity of care can be categorised as ‘upstream’ continuity (having seen the patient prior to the index consultation) and ‘downstream’ continuity (follow-up organised post-index consultation). This has been explored in a previous ReCEnT study, which found that both ‘upstream’ and ‘downstream’ continuity of care were associated with practices that universally bulk-bill [27]. In the current analysis, of consultations where registrars may choose to bulk-bill or not, association of bulk-billing with markers of continuity may suggest that registrars choose to ‘subsidise’ continuity-of-care – possibly as consultations that are congruent with the ‘principles’ of general practice [1] and with optimal patient care.

An alternative explanation for association with one of our markers of continuity (follow-up organised with another GP in the practice) is that the registrar may ‘discount’ their own contribution [8] (being trainees) if follow-up is perceived to be needed by a more senior GP and, therefore, choose not to privately-bill. This aligns with findings from the US, in which family medicine residents billed lower attendance codes compared to attending family medicine physicians, which resulted in lost revenue for teaching clinics [28, 29]. A further consideration is that continuity markers may entail frequent patient attendances/re-attendances and, so, ‘capacity to pay’ may be taken into consideration, as discussed above.

Conversely, registrars had lower odds of bulk-billing if they generated a learning goal or sought assistance from a source other than their supervisor. This may reflect a perceived increase in ‘value for money’ (for example, being able to better address the patient’s problem by seeking specialist advice), empowering the registrar to charge privately. Furthermore, in the secondary analysis, GP registrars in Term 3 were more likely to privately bill compared to registrars in Term 1, which may reflect a perceived increase in ‘value for money’ given the registrar is more experienced and closer to obtaining Fellowship.

Privately-billed consultations had greater odds of medications prescribed, pathology ordered, and imaging ordered. This may be a marker of these being more complex problems but we can also postulate that this may be due to registrars feeling obliged to give paying patients (patient-perceived) ‘value for money from the consultation’, as has been the interpretation for findings in previous studies of antibiotic prescribing [30, 31]. This is also consistent with our previous work which has found that practices in higher socioeconomic areas were associated with more instances of low-value care [32]. 

In our secondary analysis, there was a significant increase in bulk-billing rates from 2010 to 2021. Time was included in our analyses as an important covariate, considering the changes in the wider healthcare environment over time that may have influenced billing practices. However, a strong temporal trend was noted, shown in Fig. 1. This reflects nationwide Medicare statistics, which demonstrate an increase from a 79.8% bulk-billing rate in 2009-10 to 88.9% in 2020 − 21 [33]. Conversely, the average ‘out-of-pocket’ fee for patients has increased over that time, from $24.08 in 2009-10 to $42.25 in 2021 − 22 [33]. Figure 1a also shows a modest increase in bulk-billing rates from 2017 to 2020, which could possibly reflect some response to the phased re-introduction of MBS rebate indexation by the Australian Government, which began with GP bulk-billing incentives from 1 July 2017 [11]. 

### Implications for future research and clinical practice

It would appear, from our findings, that there is a financial penalty in facilitating continuity of care, in that these consultations have greater odds of being bulk-billed, thereby reducing practice and registrar income. This may be offset by CDM item numbers, which were excluded from the primary analysis (as they are generally bulk-billed due to higher Medicare rebates). However, there is insufficient evidence to suggest that these item numbers promote continuity of care or improve patient outcomes [34], and poor continuity of care continues to be reported in Australia [35]. Further research is needed to evaluate the efficacy of CDM item numbers, and policymakers could consider different funding models that promote more effective, and financially viable, continuity of care.

It was announced in the 2023–2024 federal budget that a voluntary patient registration scheme (MyMedicare), would be introduced in late 2023 with blended payment models, including additional funding for patients who are frequent hospital attenders [36]. The rollout and subsequent performance of this scheme, including patient outcomes and GP acceptability, should be subject to rigorous evaluation.

Billing decisions, and their relationship to registrar training, are important as they affect the financial viability of teaching practices as well as registrars’ incomes, which has implications for recruitment to GP vocational training of teaching practices and registrars [12, 13]. Previous Australian literature has demonstrated that teaching provides practices with only marginal net financial gain [37], with rural teaching practices incurring higher costs in teaching registrars [14], so reductions in practice income could influence their decision to employ registrars. Further, a 2023 survey of Australian medical students found that remuneration was a key barrier for students to consider a career in general practice [38]. As well as these financial considerations that may lead to less bulk-billing, there may be implications for registrars’ learning experiences. As discussed above, a previous ReCEnT study showed that continuity of care was associated with bulk-billing practices [27]. If financial and practice policy demand less bulk-billing, this could result in reduced continuity of care, which is a defining characteristic of general practice and an important component of registrar learning.

Further qualitative research is needed to explore GP registrars’ billing decisions, how this relates to their education and training, and the impacts on teaching practices. Further research should also include patient, GP supervisor, and practice manager perspectives around billing decisions.

### Strengths and limitations

Strengths were a large sample size of GP registrars with good representation from urban, regional, and rural areas, across a diverse range of demographics. There will be strong external validity for GP registrars in Australia. However, these findings will not be directly generalisable internationally, due to differing billing and funding structures. But the motivations for billing decisions suggested by the associations found in this study may be relevant to apprenticeship-like GP training regimens in countries with fee-for-service private-billing or mixed-billing remuneration.

For limitations, due to the cross-sectional nature of the study, we are unable to establish causality for these findings. Further, we were unable to exclude Department of Veterans Affairs consultations from the dataset as this categorical variable is not routinely collected by ReCEnT. However, we estimate that these consultations would account for a negligible percentage of overall encounters.

Another limitation is that we were unable to know whether the included mixed billing practices had bulk-billing policies, such as bulk-billing all pension- and concession-card holders, which may have influenced our results. Another important confounder would be whether the patient was a concession- or pension-card holder, however, this categorical variable is not routinely collected in ReCEnT. These limitations could bias the results towards higher bulk-billing rates for certain population groups, including older Australians, related to practice policies rather than registrar decision-making.

## Conclusion

Medicare billing is a complex, but important, educational issue for GPs-in-training. This is the first study, to our knowledge, that explores the billing practices of Australian GP registrars. Registrar billing decisions may, in part, reflect government bulk-billing incentives but our findings suggest other factors may contribute, including provision of affordable care recognising patient need, valuing continuity of care, and perceived patient ‘value for money’. Further qualitative research is needed to better understand how, and why, registrars make billing decisions and how these decisions impact on their training and the financial viability of teaching practices.

## Supplementary Information

Supplementary Material 1.

## Data Availability

The data that support this study cannot be publicly shared due to ethical or privacy reasons.

## Abbreviations

ACCRM: Australian College of Rural and Remote Medicine
aOR: Adjusted odds ratio
ASGS: RA–Australian Statistical Geography Standard Remoteness Area
CDM: Chronic disease management
CI: Confidence Interval
FTE: Full time equivalent
GP: General practitioner
MBS: Medicare Benefits Schedule
NESB: Non–English speaking background
RACGP: Royal Australian College of General Practitioners
ReCEnT: Registrars Clinical Encounters in Training
RTP: Regional Training Provider
SD: Standard deviation
SEIFA: IRSD–Socio–Economic Indexes for Areas–Index of Relative Socioeconomic Disadvantage
