# Supplementary material for: Australian general practice registrars’ billing patterns: a cross-sectional analysis from the Registrars Clinical Encounters in Training (ReCEnT) study
Source: BMC Health Serv Res. 2024 Nov 27;24:1483. doi: 10.1186/s12913-024-11834-y (PMC11603984; doi:10.1186/s12913-024-11834-y)
Supplement: Supplementary file 1 — Supplementary Material 1. [file 12913_2024_11834_MOESM1_ESM.docx]

**Supplementary Material**

**Table S1: Descriptive characteristics of bulk-billing versus private-billing (primary analysis, n = 3,086 GP registrars, n = 316,141 consultations, 2010-2021).**

| **Variable group** | **Variable** | **Class** | **Private** | **Bulk-billed** | **P value** |
| --- | --- | --- | --- | --- | --- |
| Time variable | Year* | mean (SD^a^) | 2016.54 (3.16) | 2016.96 (3.00) | <0.001 |
|  |  | 2010 | 2037 (2%) | 2493 (1%) | <0.001 |
|  |  | 2011 | 4968 (4%) | 6014 (3%) |  |
|  |  | 2012 | 9112 (8%) | 10094 (5%) |  |
|  |  | 2013 | 10828 (9%) | 13306 (7%) |  |
|  |  | 2014 | 9992 (8%) | 15681 (8%) |  |
|  |  | 2015 | 11345 (9%) | 19101 (10%) |  |
|  |  | 2016 | 6837 (6%) | 10904 (6%) |  |
|  |  | 2017 | 9576 (8%) | 17020 (9%) |  |
|  |  | 2018 | 15283 (13%) | 27144 (14%) |  |
|  |  | 2019 | 16187 (13%) | 28703 (15%) |  |
|  |  | 2020 | 9225 (8%) | 19448 (10%) |  |
|  |  | 2021 | 15336 (13%) | 25507 (13%) |  |
| Patient variables | Patient age group | 0-14 years | 9676 (8%) | 45085 (23%) | <0.001 |
|  |  | 35-64 years | 36857 (31%) | 41179 (21%) |  |
|  |  | 35-64 years | 58787 (49%) | 56071 (29%) |  |
|  |  | 65-74 years | 8822 (7%) | 24648 (13%) |  |
|  |  | 75+ years | 5127 (4%) | 26221 (13%) |  |
|  | Patient gender | Male | 44890 (38%) | 77433 (40%) | <0.001 |
|  |  | Female | 73669 (62%) | 114457 (60%) |  |
|  | Patient/practice status | Existing patient | 37761 (32%) | 90207 (47%) | <0.001 |
|  |  | New to registrar | 69718 (58%) | 92154 (48%) |  |
|  |  | New to practice | 11762 (10%) | 10626 (6%) |  |
|  | NESB^b^ | No | 105602 (96%) | 168236 (95%) | <0.001 |
|  |  | Yes | 4346 (4%) | 8968 (5%) |  |
|  | Aboriginal and/or Torres Strait Islander status | No | 110555 (99.3%) | 175478 (97%) | <0.001 |
|  |  | Yes | 830 (0.7%) | 4723 (3%) |  |
| Registrar variables | Registrar gender | Male | 41979 (35%) | 75434 (39%) | <0.001 |
|  |  | Female | 78747 (65%) | 119981 (61%) |  |
|  | Registrar full-time or part-time | Part-time | 29982 (25%) | 43610 (23%) | 0.005 |
|  |  | Full-time | 89688 (75%) | 149646 (77%) |  |
|  | Term | Term 1 | 45819 (38%) | 73301 (38%) | 0.16 |
|  |  | Term2 | 41729 (35%) | 69370 (35%) |  |
|  |  | Term3 | 33178 (27%) | 52744 (27%) |  |
|  | Qualified as doctor in Australia | No | 21531 (18%) | 39248 (20%) | 0.052 |
|  |  | Yes | 98207 (82%) | 154082 (80%) |  |
|  | Worked at practice previously | No | 87673 (73%) | 139731 (72%) | <0.001 |
|  |  | Yes | 31683 (27%) | 54008 (28%) |  |
|  | Has previous qualification | No previous qualifications | 68121 (57%) | 111620 (58%) | 0.45 |
|  |  | Previous qualifications, health | 15039 (13%) | 26275 (14%) |  |
|  |  | Previous qualifications, non-health | 36767 (31%) | 55766 (29%) |  |
|  | Has post-grad qualification | No | 81070 (68%) | 131469 (68%) | 0.43 |
|  |  | Yes | 39011 (32%) | 62390 (32%) |  |
|  | Has other regular medical work | No | 93493 (81%) | 152266 (80%) | 0.55 |
|  |  | Yes | 22174 (19%) | 37336 (20%) |  |
|  | Registrar age | mean (SD) | 32.71 (6.10) | 32.93 (6.31) | 0.60 |
| Practice variables | Practice size | Small | 37689 (32%) | 68036 (35%) | 0.002 |
|  |  | Large | 81545 (68%) | 125423 (65%) |  |
|  | Rurality | Major city | 64059 (53%) | 96632 (49%) | <0.001 |
|  |  | Inner regional | 42089 (35%) | 69588 (36%) |  |
|  |  | Outer regional remote | 14510 (12%) | 29170 (15%) |  |
|  | Region | Region 1 | 27282 (23%) | 46674 (24%) | <0.001 |
|  |  | Region 2 | 6699 (6%) | 9412 (5%) |  |
|  |  | Region 3 | 14901 (12%) | 24154 (12%) |  |
|  |  | Region 4 | 42348 (35%) | 57311 (29%) |  |
|  |  | Region 5 | 1839 (2%) | 2821 (1%) |  |
|  |  | Region 6 | 6694 (6%) | 21087 (11%) |  |
|  |  | Region 7 | 20963 (17%) | 33956 (17%) |  |
|  | SEIFA-IRSD^c^ | mean (SD) | 5.79 (2.64) | 5.21 (2.71) | <0.001 |
| Consultation variables | Problem status | Only existing problems | 28800 (25%) | 64462 (35%) | <0.001 |
|  |  | Only new problems | 62045 (55%) | 85848 (47%) |  |
|  |  | Both existing and new problems | 22483 (20%) | 33691 (18%) |  |
|  | Sought help any source | None | 91289 (76%) | 148201 (76%) | <0.001 |
|  |  | Supervisor | 11741 (10%) | 19097 (10%) |  |
|  |  | Other sources | 17696 (15%) | 28117 (14%) |  |
|  | Consultation duration | mean (SD) | 18.29 (8.69) | 17.83 (9.86) | <0.001 |
|  | Number of problems | mean (SD) | 1.57 (0.81) | 1.54 (0.80) | <0.001 |
|  | Medication prescribed | No | 50440 (42%) | 86374 (44%) | <0.001 |
|  |  | Yes | 70286 (58%) | 109041 (56%) |  |
|  | Imaging ordered | No | 103876 (86%) | 175200 (90%) | <0.001 |
|  |  | Yes | 16850 (14%) | 20215 (10%) |  |
|  | Pathology ordered | No | 86679 (72%) | 157612 (81%) | <0.001 |
|  |  | Yes | 34047 (28%) | 37803 (19%) |  |
|  | Referral ordered | No | 99995 (83%) | 161085 (82%) | 0.003 |
|  |  | Yes | 20731 (17%) | 34330 (18%) |  |
|  | Follow-up ordered | None | 57966 (48%) | 91330 (47%) | <0.001 |
|  |  | With themselves | 55649 (46%) | 89170 (46%) |  |
|  |  | Other GP in the practice | 7111 (6%) | 14915 (8%) |  |
|  | Learning goals generated | No | 88517 (76%) | 145280 (78%) | <0.001 |
|  |  | Yes | 27501 (25%) | 42150 (25%) |  |

*P-value on the row where class is “mean (SD)” is from a univariable logistic GEE where year is treated as a continuous variable. The p-value on the row where class is “2010” is from a univariable logistic GEE where year is treated as a categorical variable, with separate categories for each year from 2010 through 2021.

^a^ Standard deviation

^b^ Non-English speaking background

^c^ Socio-Economic Indexes for Areas – Index of Relative Socio-Economic Disadvantage

**Table S2: Missingness for the primary analysis**

| **Group** | **Variable** | **Non-Missing** | **Missing** | **% Missing** |
| --- | --- | --- | --- | --- |
| Outcome | Practice routinely bulk bills (main) | 316141 | 0 | 0.00 |
| Time | Year | 316141 | 0 | 0.00 |
| Patient | Patient age group | 312473 | 3668 | 1.16 |
|  | Patient gender | 310449 | 5692 | 1.80 |
|  | Patient/practice status | 312228 | 3913 | 1.24 |
|  | NESB | 287152 | 28989 | 9.17 |
|  | ATSI | 291586 | 24555 | 7.77 |
| Registrar | Registrar gender | 316141 | 0 | 0.00 |
|  | Registrar age | 312183 | 3958 | 1.25 |
|  | Registrar FTE | 312926 | 3215 | 1.02 |
|  | Training term/post | 316141 | 0 | 0.00 |
|  | Qualified as doctor in Australia | 313068 | 3073 | 0.97 |
|  | Worked at practice previously | 313095 | 3046 | 0.96 |
|  | Has previous qualification | 313588 | 2553 | 0.81 |
|  | Has post-grad qualification | 313940 | 2201 | 0.70 |
|  | Has other regular medical work | 305269 | 10872 | 3.44 |
| Practice | Practice size | 312693 | 3448 | 1.09 |
|  | Rurality | 316048 | 93 | 0.03 |
|  | SEIFA | 316048 | 93 | 0.03 |
|  | RTO | 316141 | 0 | 0.00 |
| Consultation | Consultation duration | 300637 | 15504 | 4.90 |
|  | Problem status | 297329 | 18812 | 5.95 |
|  | Number of problems | 316080 | 61 | 0.02 |
|  | Sought assistance | 316141 | 0 | 0.00 |
| Consultation outcome | Medication prescribed | 316141 | 0 | 0.00 |
|  | Imaging ordered | 316141 | 0 | 0.00 |
|  | Pathology ordered | 316141 | 0 | 0.00 |
|  | Referral ordered | 316141 | 0 | 0.00 |
|  | Follow-up ordered | 316141 | 0 | 0.00 |
|  | Learning goals generated | 303448 | 12693 | 4.01 |

**Table S3: Descriptive characteristics of bulk-billing versus private-billing (secondary analysis, n = 3,850 GP registrars, n = 548,066 consultations, 2010-2021).**

| **Variable group** | **Variable** | **Class** | **Private** | **Bulk-billed** | **P value** |
| --- | --- | --- | --- | --- | --- |
| Time variable | Year* | mean (SD^a^) | 2016.75 (3.12) | 2017.71 (2.82) | 0.019 |
|  |  | 2010 | 2074 (1%) | 3172 (0.8%) | <0.001 |
|  |  | 2011 | 5199 (4%) | 7868 (2%) |  |
|  |  | 2012 | 9459 (7%) | 14537 (4%) |  |
|  |  | 2013 | 11290 (8%) | 19384 (5%) |  |
|  |  | 2014 | 12047 (8%) | 23698 (6%) |  |
|  |  | 2015 | 12892 (9%) | 28008 (7%) |  |
|  |  | 2016 | 8339 (6%) | 16967 (4%) |  |
|  |  | 2017 | 11711 (8%) | 36996 (9%) |  |
|  |  | 2018 | 17920 (13%) | 59457 (15%) |  |
|  |  | 2019 | 19216 (13%) | 62144 (15%) |  |
|  |  | 2020 | 12683 (9%) | 66823 (16%) |  |
|  |  | 2021 | 19578 (14%) | 66544 (16%) |  |
| Patient variables | Patient age group | 0-14 years | 12218 (9%) | 77464 (19%) | <0.001 |
|  |  | 35-64 years | 44409 (32%) | 105317 (26%) |  |
|  |  | 35-64 years | 67687 (48%) | 134653 (34%) |  |
|  |  | 65-74 years | 10258 (7%) | 42397 (11%) |  |
|  |  | 75+ years | 6174 (4%) | 41635 (10%) |  |
|  | Patient gender | Male | 53067 (38%) | 160999 (40%) | <0.001 |
|  |  | Female | 86864 (62%) | 238210 (60%) |  |
|  | Patient/practice status | Existing patient | 44484 (32%) | 180182 (45%) | <0.001 |
|  |  | New to registrar | 81683 (58%) | 194866 (49%) |  |
|  |  | New to practice | 14478 (10%) | 25728 (6%) |  |
|  | NESB^b^ | No | 122761 (95%) | 323631 (90%) | <0.001 |
|  |  | Yes | 6414 (5%) | 36356 (10%) |  |
|  | Aboriginal and/or Torres Strait Islander status | No | 130016 (99.3%) | 360350 (98%) | <0.001 |
|  |  | Yes | 963 (0.7%) | 8266 (2%) |  |
| Registrar variables | Registrar gender | Male | 50662 (36%) | 165305 (41%) | <0.001 |
|  |  | Female | 91744 (64%) | 240176 (59%) |  |
|  | Registrar FT or PT | Part-time | 33678 (25%) | 85862 (22%) | 0.025 |
|  |  | Full-time | 103365 (75%) | 298008 (78%) |  |
|  | Term | Term 1 | 52558 (37%) | 152726 (38%) | 0.008 |
|  |  | Term2 | 49049 (34%) | 143993 (36%) |  |
|  |  | Term3 | 40801 (29%) | 108879 (27%) |  |
|  | Qualified as doctor in Australia | No | 24590 (17%) | 75693 (19%) | 0.10 |
|  |  | Yes | 116216 (83%) | 322961 (81%) |  |
|  | Worked at practice previously | No | 102603 (75%) | 296335 (76%) | <0.001 |
|  |  | Yes | 35106 (25%) | 92827 (24%) |  |
|  | Has previous qualification | No previous qualifications | 79453 (56%) | 226016 (57%) | 0.53 |
|  |  | Previous qualifications, health | 17712 (13%) | 52327 (13%) |  |
|  |  | Previous qualifications, non-health | 43851 (31%) | 120624 (30%) |  |
|  | Has post-grad qualification | No | 95826 (68%) | 272118 (68%) | 0.62 |
|  |  | Yes | 45346 (32%) | 126712 (32%) |  |
|  | Has other regular medical work | No | 108225 (81%) | 316302 (83%) | 0.53 |
|  |  | Yes | 24951 (19%) | 63922 (17%) |  |
|  | Registrar age | mean (SD) | 32.67 (6.04) | 32.65 (6.22) | 0.082 |
| Practice variables | Practice size | Small | 42908 (31%) | 162195 (42%) | <0.001 |
|  |  | Large | 93541 (69%) | 221586 (58%) |  |
|  | Rurality | Major city | 77856 (55%) | 256601 (63%) | 0.011 |
|  |  | Inner regional | 48442 (34%) | 108039 (27%) |  |
|  |  | Outer regional remote | 16035 (11%) | 40756 (10%) |  |
|  | Region | Region 1 | 31645 (22%) | 67783 (17%) | <0.001 |
|  |  | Region 2 | 7188 (5%) | 14041 (3%) |  |
|  |  | Region 3 | 17226 (12%) | 29044 (7%) |  |
|  |  | Region 4 | 49069 (34%) | 112988 (28%) |  |
|  |  | Region 5 | 2296 (2%) | 4221 (1%) |  |
|  |  | Region 6 | 9828 (7%) | 109661 (27%) |  |
|  |  | Region 7 | 25156 (18%) | 67860 (17%) |  |
|  | SEIFA-IRSD^c^ | mean (SD) | 5.84 (2.65) | 5.22 (2.84) | <0.001 |
| Consultation variables | Problem status | Only existing problems | 34268 (26%) | 133372 (35%) | <0.001 |
|  |  | Only new problems | 73664 (55%) | 184700 (48%) |  |
|  |  | Both existing and new problems | 25915 (19%) | 66080 (17%) |  |
|  | Sought help any source | None | 108003 (76%) | 311086 (77%) | <0.001 |
|  |  | Supervisor | 13644 (10%) | 37572 (9%) |  |
|  |  | Other sources | 20761 (15%) | 56940 (14%) |  |
|  | Consultation duration | mean (SD) | 18.27 (8.76) | 17.43 (9.86) | <0.001 |
|  | Number of problems | mean (SD) | 1.56 (0.80) | 1.50 (0.78) | <0.001 |
|  | Medication prescribed | No | 60040 (42%) | 188311 (46%) | <0.001 |
|  |  | Yes | 82368 (58%) | 217287 (54%) |  |
|  | Imaging ordered | No | 122844 (86%) | 363495 (90%) | <0.001 |
|  |  | Yes | 19564 (14%) | 42103 (10%) |  |
|  | Pathology ordered | No | 102634 (72%) | 325131 (80%) | <0.001 |
|  |  | Yes | 39774 (28%) | 80467 (20%) |  |
|  | Referral ordered | No | 117982 (83%) | 335307 (83%) | <0.001 |
|  |  | Yes | 24426 (17%) | 70291 (17%) |  |
|  | Follow-up ordered | None | 69502 (49%) | 192467 (47%) | <0.001 |
|  |  | With themselves | 64766 (45%) | 187082 (46%) |  |
|  |  | Other GP in the practice | 8140 (6%) | 26048 (6%) |  |
|  | Learning goals generated | No | 104972 (77%) | 306445 (78%) | <0.001 |
|  |  | Yes | 31862 (23%) | 84358 (22%) |  |

*P-value on the row where class is “mean (SD)” is from a univariable logistic GEE where year is treated as a continuous variable. The p-value on the row where class is “2010” is from a univariable logistic GEE where year is treated as a categorical variable, with separate categories for each year from 2010 through 2021.

^a^ Standard deviation

^b^ Non-English speaking background

^c^ Socio-Economic Indexes for Areas – Index of Relative Socio-Economic Disadvantage

**Table S4: Missingness for the secondary analysis**

| **Group** | **Variable** | **Non-Missing** | **Missing** | **% Missing** |
| --- | --- | --- | --- | --- |
| Outcome | Practice routinely bulk bills (sensitivity) | 548006 | 0 | 0.00 |
| Time | Year | 548006 | 0 | 0.00 |
| Patient | Patient age group | 542212 | 5794 | 1.06 |
|  | Patient gender | 539140 | 8866 | 1.62 |
|  | Patient/practice status | 541421 | 6585 | 1.20 |
|  | NESB | 489162 | 58844 | 10.74 |
|  | ATSI | 499595 | 48411 | 8.83 |
| Registrar | Registrar gender | 547887 | 119 | 0.02 |
|  | Registrar age | 536138 | 11868 | 2.17 |
|  | Registrar FTE | 520913 | 27093 | 4.94 |
|  | Training term/post | 548006 | 0 | 0.00 |
|  | Qualified as doctor in Australia | 539460 | 8546 | 1.56 |
|  | Worked at practice previously | 526871 | 21135 | 3.86 |
|  | Has previous qualification | 539983 | 8023 | 1.46 |
|  | Has post-grad qualification | 540002 | 8004 | 1.46 |
|  | Has other regular medical work | 513400 | 34606 | 6.31 |
| Practice | Practice size | 520230 | 27776 | 5.07 |
|  | Rurality | 547729 | 277 | 0.05 |
|  | SEIFA | 547536 | 470 | 0.09 |
|  | RTO | 548006 | 0 | 0.00 |
| Consultation | Consultation duration | 523402 | 24604 | 4.49 |
|  | Problem status | 517999 | 30007 | 5.48 |
|  | Number of problems | 547907 | 99 | 0.02 |
|  | Sought assistance | 548006 | 0 | 0.00 |
| Consultation outcome | Medication prescribed | 548006 | 0 | 0.00 |
|  | Imaging ordered | 548006 | 0 | 0.00 |
|  | Pathology ordered | 548006 | 0 | 0.00 |
|  | Referral ordered | 548006 | 0 | 0.00 |
|  | Follow-up ordered | 548005 | 1 | 0.00 |
|  | Learning goals generated | 527637 | 20369 | 3.72 |
